# Targeting glucose metabolism for treatment of COVID-19

**DOI:** 10.1038/s41392-021-00532-4

**Published:** 2021-03-06

**Authors:** Amin Ardestani, Zahra Azizi

**Affiliations:** 1 Centre for Biomolecular Interactions Bremen, University of Bremen, Bremen, Germany; 2 Department of Molecular Medicine, School of Advanced Technologies in Medicine, Tehran University of Medical Sciences, Tehran, Iran

**Keywords:** Endocrine system and metabolic diseases, Physiology

A recent study published in *Cell Metabolism* by Codo et al.^1^ shows that metabolic rewiring of human monocytes by SARS-CoV-2 infection cultured under high glucose highly induces viral replication and cytokine production compromising T-cell response and function. Ultimately, this triggers lung epithelial cell death and, mechanistically, provides an explanation why people with diabetes might be more susceptible to develop severe COVID-19.

COVID-19 caused by the SARS-CoV-2 represents a significant public health burden; its high prevalence worldwide has dramatic consequences on the society at all levels. Obesity and related metabolic diseases particularly type 2 diabetes (T2D) are linked with more devastating COVID-19 disease courses.^2^ Recent epidemiological data demonstrate that rate of COVID-19 induced morbidity as well as mortality is markedly higher in people with obesity and T2D.^2^ Consistently, multiple clinical studies including a retrospective examination of a large cohort of COVID-19 patients have shown that poorly controlled blood glucose levels are associated with highest COVID-19 mortality rates.^2^ This suggests that COVID-19 pathogenesis and the regulation of metabolism in the host are intimately connected. Little is known about the molecular mechanisms of deregulated glucose metabolism, especially under insulin therapy, that determine vulnerability of cells and organs in a diabetic environment to a severe SARS-CoV-2 disease outcome.

Viruses alter the host cell metabolism in order to make optimal conditions for their rapid and efficient replication and spread. One key particular example is the enhanced uptake of important nutrients such as glucose to support metabolic signaling, i.e., aerobic glycolysis, a primary pathway of glucose metabolism and its by-products for biosynthetic reactions. Abnormal glucose metabolism primarily caused by impaired insulin secretion and/or action is a characteristic feature of T2D and affects several tissues highly important for the regulation of whole body metabolism, such as liver, adipocytes, muscle, pancreatic islets, and immune cells. The increased glucose metabolism imposed by sustained hyperglycemia may enhance SARS-CoV-2’s entry and subsequent replication, as well as an exacerbated immune response in individuals with diabetes. Thus, a disrupted glucose metabolism and metabolic derangement in diabetes may be an intrinsic cellular strategy that favors SARS-CoV-2 pathogenesis. In this context, Codo et al.^1^ explored the molecular response of SARS-CoV-2 infected human monocytes under diabetic condition. The authors initially show that SARS-CoV-2 efficiently infects peripheral blood monocytes, upregulates angiotensin-converting enzyme 2 (ACE2), a key SARS-CoV-2’s receptor and highly induced proinflammatory cytokines such as TNF-α, IL-1β, and IL-6. This is consistent with the altered innate immune response and excessive inflammatory cytokine production, the so-called “cytokine storm” observed in severe COVID-19.^1^ Notably, dose-dependent increase in glucose concentrations potentiate SARS-CoV-2 replication as well as ACE2 upregulation and cytokine production in monocytes suggesting elevated glucose as a principal promoter of virus replication and inflammatory response. SARS-CoV-2 directly induces glycolysis in monocytes, which is in line with the enrichment of glycolytic genes and metabolic remodeling observed by single-cell RNA sequencing (RNA-seq) of lung monocyte from COVID-19 patients.^1^

**Fig. 1:**
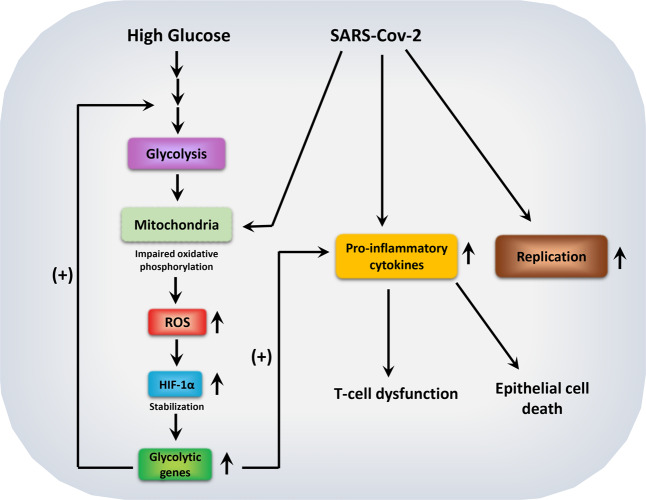
Proposed model of how glucose induced metabolic reviewing potentiates SARS-Cov-2 replication and cytokine production. In metabolically stressed monocytes (cultured under high glucose condition), SARS-CoV-2 infection enhances glycolysis by aberrant production of mitochondrial ROS which leads to activation of transcription factor hypoxia-inducible factor-1α (HIF-1α) to ensure its rapid replication. Such increased glucose metabolism through aerobic glycolysis fosters viral replication and cytokine production compromising T-cell response and function as well as induces lung epithelial cell death

To understand the biochemical mechanism required for SARS-CoV-2’ replication and its impact in monocytes, Codo et al.^1^ provided conclusive evidences that glycolytic flux is indispensable for SARS-CoV-2’s impact. Through well-designed experiments, the authors show that inhibition of glycolysis by 2-deoxy-d-glucose (2-DG) as well as inhibition of glycolytic enzymes 6-phospho-fructo-2-kinase/fructose-2,6-bisphosphatase-3 (PFKFB3), a positive regulator of phosphofructokinase-1 (PFK1) as well as lactate dehydrogenase A (LDH-A) abolishes viral replication and cytokine response placing glycolysis as a key upstream event during SARS-CoV-2 pathogenesis, although critical glycolytic intermediate/final product(s) mediating such effect was not determined. Similarly, 2-DG blocks SARS-CoV-2 replication in a colon epithelial carcinoma cell line.^3^ The metabolic transcription factor hypoxia-inducible factor-1α (HIF-1α) is a master regulator of glycolysis and HIF-1α levels and activity as well as target genes are strongly induced in SARS-CoV-2 infected monocytes consistent with elevated HIF-1α in blood monocytes isolated from critical COVID-19 patients.^1^ Codo et al. further identified that HIF-1α inhibition or stabilization blocks or exacerbates HIF-1α target genes, viral replication and ACE2 and cytokine expression, indicating that HIF-1α is essential for elevated glycolysis and subsequent inflammatory responses. The authors further offer mechanistic insight into how HIF-1α is stabilized under increased glucose condition. Through a series of biochemical experiments, they show that an increase in mitochondrial reactive oxygen species (ROS) due to impaired oxidative metabolism is responsible for HIF-1α stabilization and the proinflammatory state in SARS-CoV-2 infected monocytes^1^ (Fig. 1).

Finally, the authors investigated whether metabolic remodeling and altered immune state of monocytes potentiated by high glucose during SARS-CoV-2 infection impairs T cell function and response. Conditioned media obtained from metabolically primed SARS-CoV-2 infected monocytes compromise CD4 or CD8 T-cell proliferation and also induce lung epithelial cell death.^1^ Inhibition of HIF-1α, as well as neutralization of ROS or IL-1β antagonize such effects and restore T-cell function or lung epithelial cell survival, which suggests that increased glucose metabolism through aerobic glycolysis and subsequent cytokine production in monocytes could further deteriorate neighboring cells in a paracrine fashion under pro-diabetic conditions in COVID-19 patients (Fig. 1).

The cellular energy status is instrumental for coordinating inflammatory responses as the host cellular metabolism is critical for anti-virulence during infection. For example, the proinflammatory cytokine IL-1β is increased in people with COVID-19. IL-1β processing is regulated through generation of 3-phosphoglycerate, a by-product of glycolysis suggesting a direct link to glucose metabolism. Furthermore, the inhibition of glycolysis with 2-DG was shown to halt IL-1β induction and to protect against LPS-induced sepsis in mice.^4^ This together with Codo et al.‘s findings indicate that SARS-CoV-2 replication and cellular host response are promoted by metabolic rewiring including a shift to aerobic glycolysis. Similarly, proinflammatory cytokines induced by Influenza A virus (IAV) infection is regulated by glucose metabolism reprogramming^5^ emphasizing the importance of glucose metabolism in virus-induced cytokine storm.

Dysregulated glucose metabolism in people with diabetes may explain the increased susceptibility to SARS-CoV-2 and why uncontrolled diabetes can lead to excessive adaptive immune reactions in patients with critical COVID-19 symptoms. Thus, targeting glucose metabolism may offer new effective antiviral and organ-supportive approaches for the treatment of COVID-19, especially in individuals with metabolic diseases.
